# Temporal dynamics of gene and protein signatures following volumetric muscle loss

**DOI:** 10.3389/fcell.2025.1606609

**Published:** 2025-07-03

**Authors:** Ishita Jain, Beu P. Oropeza, Caroline Hu, Gladys Chiang, Sree Aravindan, Renato Reyes, Daniel Yuhang Li, Paul Cheng, Ngan F. Huang

**Affiliations:** ^1^ Department of Cardiothoracic Surgery, Stanford University, Stanford, CA, United States; ^2^ Stanford Cardiovascular Institute, Stanford University, Stanford, CA, United States; ^3^ Center for Tissue Regeneration, Repair and Restoration, Veterans Affairs Palo Alto Healthcare System, Palo Alto, CA, United States; ^4^ Division of Cardiovascular Medicine, Stanford University, Stanford, CA, United States; ^5^ Cardiovascular Institute, Stanford University, Stanford, CA, United States; ^6^ Department of Chemical Engineering, Stanford University, Stanford, CA, United States

**Keywords:** volumetric muscle loss (VML), RNA sequencing, proteomics, gene signature, muscle regeneration

## Abstract

**Introduction:**

Volumetric muscle loss (VML) is characterized by permanent tissue impairment resulting from critically-sized muscle loss. We performed time-series transcriptomic and proteomic analyses to reveal key mediators of irreversible pathological remodeling after induction of VML in mice.

**Methods:**

The dynamics of gene and protein expression patterns were analyzed for up to 3 weeks after muscle injury.

**Results:**

RNA Sequencing revealed transcriptional patterns that show rapid upregulation or downregulation shortly after injury, among which a subset of genes failed to return to pre-injury levels within 3 weeks after VML. Time-series analysis revealed gene clusters with sustained upregulation after 3 weeks, including those associated with extracellular matrix remodeling and inflammation, whereas the gene clusters having sustained downregulation were associated with mitochondrial function and metabolism. We further identified *SPI1* and *SP1* as novel molecular mediators of the pathological remodeling process.

**Discussion:**

This work demonstrates the utility of time-series analysis to reveal dysregulated pathways in the setting of VML.

## Graphical Abstract

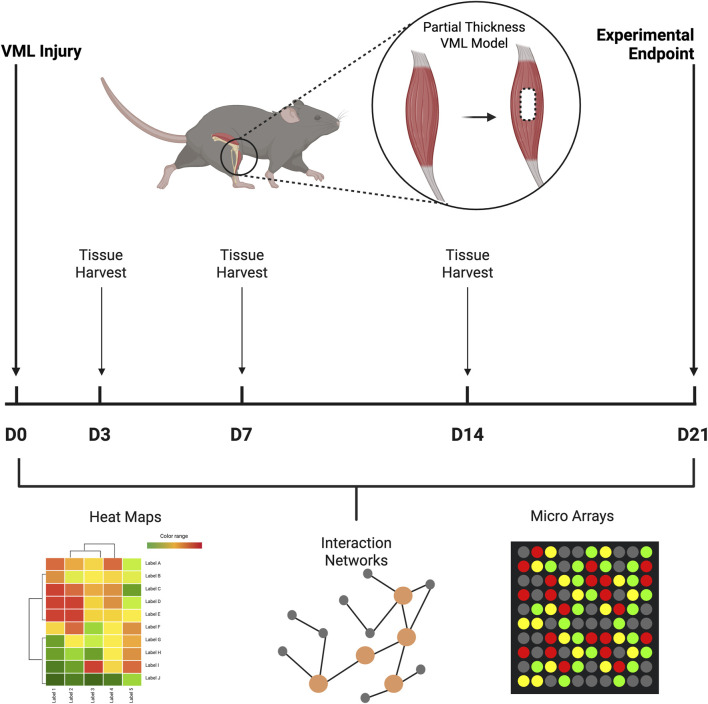

## Introduction

Volumetric muscle loss (VML) is characterized by the irreversible loss of muscle function due to the traumatic loss of a critically sized volume of muscle tissue, leading to life-long disability and cosmetic deformities (Garg et al., 2015). The incidence of VML resulting from military interventions or civilian accidents is rising (Owens et al., 2008). Standard surgical treatment of VML using muscle flap transfer or tissue debridement results in donor site morbidity or functional deficiency (Lin et al., 2007; Klinkenberg et al., 2013). Other potential treatments under experimental investigation include cell, biomaterials, and/or gene therapies, often in conjunction with rehabilitation (Lin et al., 2004; Sicari et al., 2014; Quarta et al., 2017; Nakayama et al., 2018; Rao et al., 2018; Nakayama et al., 2019; Chan et al., 2023). However, efforts to develop effective therapeutics to treat VML are hampered by the incomplete mechanistic understanding underlying pathological remodeling after VML.

Small-sized muscle injuries can reversibly recover with time, but those exceeding a critical size result in permanent muscle impairment, thereby suggesting the dysregulation of intrinsic muscle healing processes associated with large defects (Westman et al., 2021). Multi-omics approaches, including transcriptomics and proteomics, have emerged as powerful tools for unraveling the molecular intricacies of injuries, such as VML (Westman et al., 2021; Na et al., 2023; Liu et al., 2024a; Liu et al., 2024b; Sanches et al., 2024). Here, we studied the dynamic temporal changes in gene and protein expression patterns driving impaired VML recovery that would be otherwise overlooked using conventional differential expression analytical methods (Howard et al., 2020; Wang and Zhou, 2022; Careccia et al., 2023). Temporal expression pattern analysis has been applied in other settings, such as tissue development and disease progression over time, leading to a new fundamental understanding of dynamics pathways involved in biological processes and drug targets (Calvano et al., 2005; Androulakis et al., 2007). However, temporal expression pattern analysis has yet to be applied to studying tissue remodeling after VML.

Here, we performed time-series analyses of transcriptomic and proteomic changes associated with VML in a mouse model, focusing on the dynamics of gene and protein expression patterns for up to 3 weeks after muscle injury. We identified signaling pathways associated with temporal expression patterns that fail to restore to pre-injury levels within 3 weeks after VML, including genes with sustained upregulation or downregulation. Using temporal expression analysis, we identified *SP1* as a novel molecular mediator of dysregulated muscle recovery after VML and elucidated pro-inflammatory and extracellular matrix (ECM) remodeling pathways mediating the remodeling process. These insights pave the way for the future development of new targets that promote muscle regeneration and functional recovery of traumatically injured muscle.

## Materials and methods

### Mouse VML model

We utilized a well-established mouse partial-thickness VML model, in which 40% of the tibialis anterior muscle in C57BL/6 (8-week-old male) mice was bilaterally excised, based on our previous work (Quarta et al., 2017; Nakayama et al., 2019). At time points of 3, 7, 14, and 21 days after the induction of VML, the tibialis anterior muscles were explanted for protein and RNA extraction (*n* = 5–6 samples per time point). All animal studies were performed with the approval of the Institutional Animal Care and Use Committee of the Veterans Affairs Palo Alto Healthcare System.

### RNA extraction and bulk RNA sequencing

Explanted muscle samples were stabilized in RNAlater (Invitrogen) at −80°C prior to RNA extraction. Tissues were mechanically homogenized in Trizol following routine RNA isolation methods. In brief, the lysed tissues were treated with chloroform, allowing for a biphasic separation of the RNA content, followed by RNA precipitation in isopropyl alcohol. The purified RNA was then resuspended in RNAase-free water. Bulk RNA sequencing was performed by Novogene Corporation, in which messenger RNA was purified from total RNA using poly-T oligo-attached magnetic beads. cDNA was synthesized, followed by non-directional library preparation for sequencing on a Novaseq 6000 instrument. Raw data (raw reads) of fastq format were filtered based on quality metrics relating to adapter content, poly-N reads, and low-quality reads. All the downstream analyses were based on clean, high-quality data. The reads were aligned to a reference genome using Hisat v2.0.5. FeatureCounts v1.5.0-p3 was used to count the reads mapped to each gene. The FPKM (fragments per kilobase of transcript per million mapped reads) of each gene was calculated based on the length of the gene and the reads count mapped to this gene.

### RNA sequencing analysis

The samples reflecting different time points were time-aligned for a better structure of the count data. The FPKM (Fragments Per Kilobase of exon per Million) counts were normalized and then log transformed. Linear modeling as a function of time was performed using the limma package in R. Pairwise comparisons were performed for all time points. The differentially expressed genes for pairwise combinations were consolidated in a single file with all the raw counts. These raw expression values of the differentially expressed genes were entered into the Mfuzzy package for time-series analysis (Hathaway and Bezdek, 1985; Kumar and Futschik, 2007; Oh and Li, 2021). The number of clusters was based on the different behaviors observed and the ease of separation of biological relevance. For each time cluster, a core-cluster gene list was obtained for further analysis based on the enrichment of each gene in each cluster, ensuring only the genes that followed the temporal cluster pattern were chosen for further analysis. Using this gene list, gene enrichment analysis was performed using the EnrichR website. The enrichment p-values and odds ratio were quantified, and RStudio was used to generate plots (ggplot2). The core-cluster genes were also used as input in the NetworkAnalyst website to construct transcription factor (TF)-gene networks. Statistical analysis was performed by the limma package in R that uses Benjamini–Hochberg correction to calculate the adjusted P value for each comparison. Statistical significance was accepted at P < 0.05. The VML signature was obtained by comparing our data to existing datasets for clinical (PRJNA491748) and mouse toxin-induced injury (GSE138826), respectively. For the clinical dataset, the differentially expressed genes (DEGs) for the analysis of heathy compared to patients with critical illness myopathy were compared to the DEGs for day 21 VML injury versus no injury control groups. For the mouse toxin injury data, DEGs were obtained for Day 21 post toxin injury versus healthy control and compared to our day 21 post VML versus no injury control groups.

### Protein extraction and proteomic analysis

Tissue samples were homogenized in a solution consisting of RIPA lysis buffer (Thermo Fisher Scientific) with protease inhibitors, incubated on ice, and then centrifuged to separate tissue components. Total protein concentration was measured using a Bicinchoninic acid protein assay (Thermo Fisher Scientific). RayBiotech Corporation performed a mouse proteomic panel of 32 cytokines (AAM-CYT-G2). In brief, equally loaded protein lysates were incubated onto membranes, followed by incubation of the membranes with a biotinylated detection antibody cocktail, followed by horse radish peroxidase-conjugated streptavidin. The cytokine arrays were imaged for chemiluminescence intensity, followed by quantification by densitometry. Proteins that exhibited sustained upregulation or sustained downregulation were fed into the STRING database to understand protein interactions. Statistical analysis was performed by repeated measures ANOVA with Tukey *post hoc* testing. Statistical significance was accepted at P < 0.05.

### Quantitative PCR (qPCR)

To perform gene expression analysis, total RNA underwent reverse transcription using the First Strand cDNA synthesis protocol based on the manufacturer’s instructions (Invitrogen). The primers used to qPCR consisted of Nmrk, Pfkfb3, Bdh1, Sln, Chrna1, Krt18, and GAPDH (Applied Biosystems, Foster City, CA). The qPCR was performed on the QuantStudio Real-Time PCR system (Fisher Scientific) for 40 cycles. The ΔΔCt method of analysis was performed, normalized to GAPDH, and then expressed as relative normalized fold change (*n* = 4–5) (Huang et al., 2010). Statistical analysis of two groups was performed by an unpaired t-test. Statistical significance was accepted at P < 0.05.

### Tissue histology

Mouse tibialis anterior muscle was collected from healthy (no injury control) mice or 21 days after induction of VML. Tissue cross-sections underwent immunofluorescence staining for SP1 antibody (Abcam). For each animal, four images were acquired by a Keyence fluorescence microscope (BZ-X710) and then quantified using ImageJ software for mean intensity (*n* = 6). Statistical analysis of two groups was performed by an unpaired t-test. Statistical significance was accepted at P < 0.05.

## Results

### Transcriptomics analysis reveals gene clusters with distinct patterns of temporal expression

**FIGURE 1:**
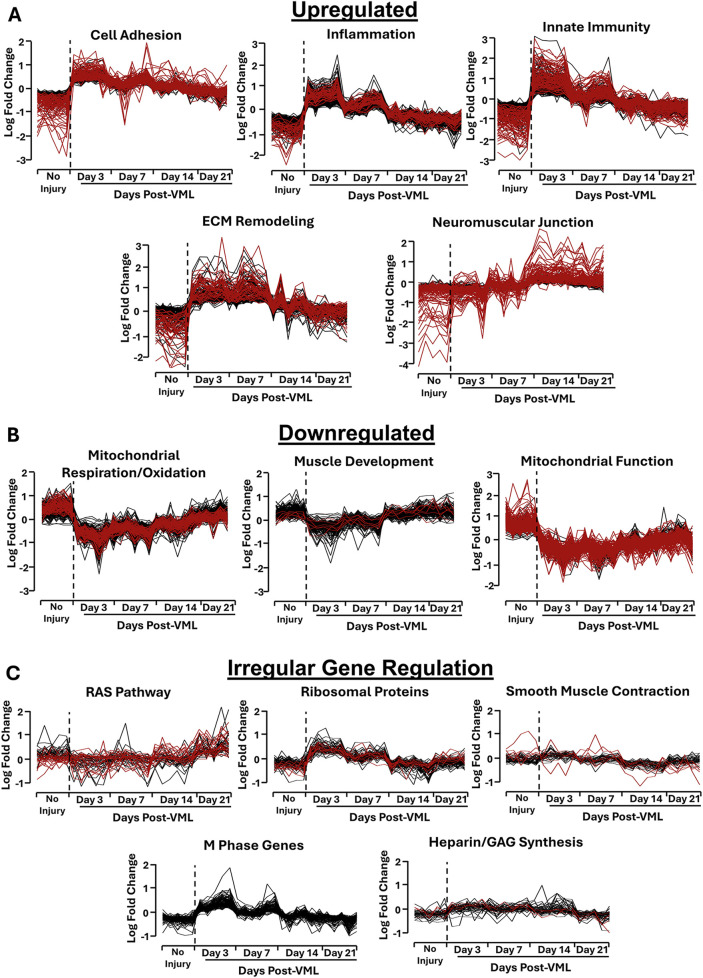
Temporal clusters identified from transcriptomics profiling of tibialis anterior muscle tissue samples after VML injury using time-series analysis. **(A,B)** Gene clusters that represent upregulation **(A)** or downregulation **(B)** after injury. **(C)** Gene clusters that exhibit irregular temporal expression patterns following VML. Data generated using Mfuzzy package in R. The black lines within each line plot denote genes that were differentially expressed at one or more time points. The red lines indicate genes that showed sustained upregulation after 21 days, compared to the control (non-injured) samples (P < 0.05).

Bulk RNA sequencing was performed to elucidate the temporal dynamics of transcriptomics changes in the tibialis anterior muscle samples at time points up to 3 weeks following VML in a mouse model. The VML samples were compared to non-injured (healthy control) tibialis anterior muscle tissue as a basis for comparison. We identified approximately 2,500 genes that were differentially expressed at least at two time points. Time series analysis of genes showing differential expression at least at two time points revealed a total of 13 temporal clusters (Figure 1), in which 5 clusters were associated with rapid upregulation immediately after injury, and a subset of those (in red) remained upregulated by >2.2 fold change after 21 days. Based on gene ontology analysis, the clusters with rapid upregulation were categorized as ECM Remodeling, Inflammation, Neuromuscular Junction, Cell Adhesion, and Innate Immunity (Figure 1A). In contrast, 3 gene clusters shared a similar pattern of rapid downregulated soon after injury, where a subset of those remained downregulated by >2.2 fold-change for 21 days. These clusters showing downregulation were categorized as Mitochondrial Function, Mitochondrial Respiration/Oxidation, and Muscle Development (Figure 1B). Five additional gene clusters had non-uniform gene expression patterns, comprising mixed patterns of temporal gene upregulation and downregulation. These clusters were assigned as RAS Pathway; Ribosomal Proteins, Smooth Muscle Contractions; M Phase Genes; and Heparin Sulfate/Glycosaminoglycan (GAG) Synthesis (Figure 1C).

### ECM remodeling cluster shows sustained upregulated expression and cell cycle transcription factor-gene networks

**FIGURE 2:**
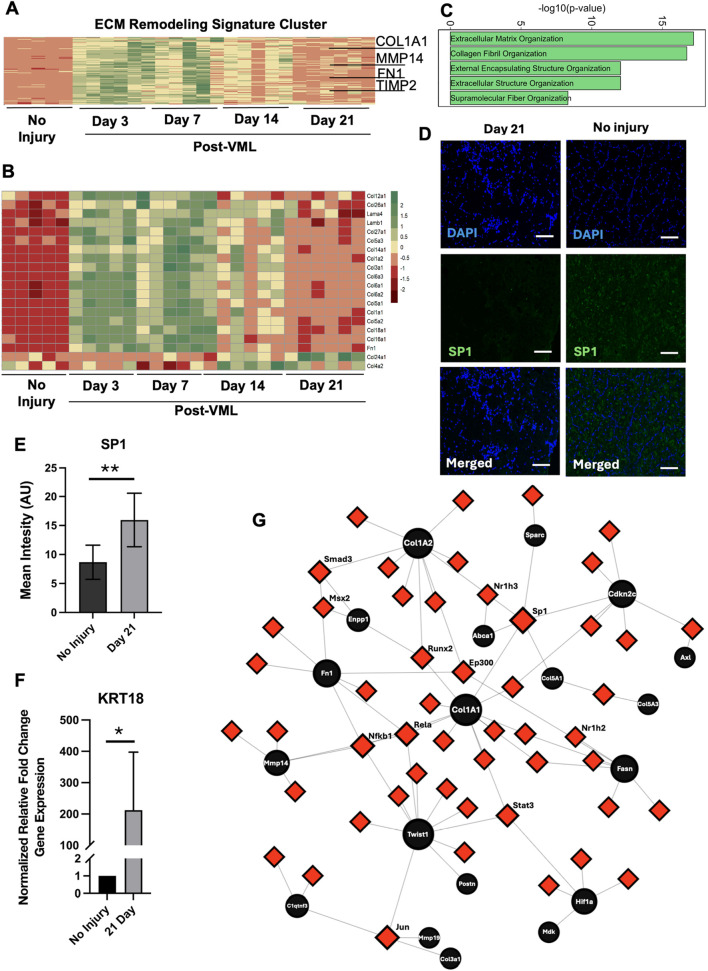
ECM remodeling cluster shows sustained upregulated expression and cell cycle transcription factor-gene networks. **(A)** Heatmap of all genes belonging to the ECM remodeling cluster. **(B)** Heatmap of all ECMs found to be differentially expressed. **(C)** Gene enrichment bar plot of genes in the ECM remodeling cluster. **(D)** Immunofluorescence staining of SP1 in the area of VML after 21 days, compared to no injury control muscle samples. **(E)** Quantification of SP1 mean fluorescence intensity in arbitrary units (AU, *n* = 6, **P < 0.01) **(F)** qPCR gene expression validation of *KRT18* (*P < 0.05, *n* = 4–5). **(G)** Gene-Transcription factor networks of genes (black circles) in the ECM remodeling cluster and their connection with any known transcription factors. Scale bar: 100 µm.

The ECM remodeling cluster, which included genes like *COLL1A1*, *FN1*, and *MMP14*, showed sharp gene upregulation after VML injury (>14-fold change at day 3, compared to day 0). A progressive decline followed this in expression levels, in which a subset did not normalize to pre-injury levels after 21 days (Figures 1A, 2A). Some key genes that remained upregulated on day 21 within this cluster included ECMs associated with fibrosis, such as *COL1A1* and *FN1*, and ECM remodeling genes, such as *TIMP2* and *MMP14*. Although 15 collagen genes within the cluster shared the temporal pattern of having a sharp upregulation after VML injury, *COL4A2* and *COL24A1* were notably not upregulated until day 14 and remained elevated until day 21 (Figure 2B). This finding is consistent with collagen IV being associated with elevated levels in VML and contributes to fibrotic scar content (Hoffman et al., 2022). The overall identity of this cluster was identified using Gene Ontology, where the gene sets included Collagen Fibril Organization (P = 3.84 × 10^−20^), Extracellular Matrix Organization (P = 5.81 × 10^−21^), and Extracellular Structure Organization (P = 2.97 × 10^−15^) (Figure 2C). Among the ECM-related genes, *SP1* is a transcription factor that is involved in ECM remodeling and drives fibrosis (Feng et al., 2023). Since the role of *SP1* in the VML is largely unknown, we performed immunofluorescence analysis of SP1 protein expression, showing that SP1 is significantly more abundant after 21 days after VML, compared to healthy no-injury controls (**P < 0.01, Figures 2D,E). Furthermore, DEGs representing the ECM cluster were further verified by qPCR. For example, the differentially upregulated cytoskeletal gene, *KRT18* (Keratin 18, P < 0.0001), was reflected by a >200-fold upregulation after 21 days based on qPCR, compared to the no-injury control group (*P < 0.05, Figure 2F). Together, these findings suggest sustained fibrosis and ECM remodeling in the VML injury after 21 days, which is consistent with previous reports (Crum et al., 2022).

To further explore the mechanistic interactions of the gene pattern, we identified key transcription factors mediating the process using NetworkAnalyst to create transcription factor-gene networks. This analysis revealed that *COL1A1*, which had a 5.6-fold change on day 21 vs. control), was one the most connected nodes (black circles) in the network and was regulated by many transcription factors (red squares) such as *SP1* (Figure 2G). The closest neighbors of *COL1A1* were *CDKN2C* and *TWIST1*, both of which were upregulated with >3-fold change on day 3, compared to day 0. Since *CDKN2C* is a negative regulator of cell proliferation, its close distance to *COL1A1* suggests a potential role of ECM remodeling-mediated cell cycle regulation (Leone et al., 2008). Furthermore, *TWIST1* is a transcription factor generally found in skeletal muscle that triggers muscle atrophy (Parajuli et al., 2018), so the increased expression of ECM-related genes may be indicative of muscle atrophy, which often accompanies VML (Greising et al., 2017). Together, these findings based on genes with a sustained upregulation suggest potentially meaningful dynamic interactions among ECM remodeling, cell cycle modulation, and atrophy genes that limit muscle repair and regeneration after VML.

### Inflammatory signaling cluster shows sustained upregulated expression and *SPI1* transcription factor-gene networks

**FIGURE 3:**
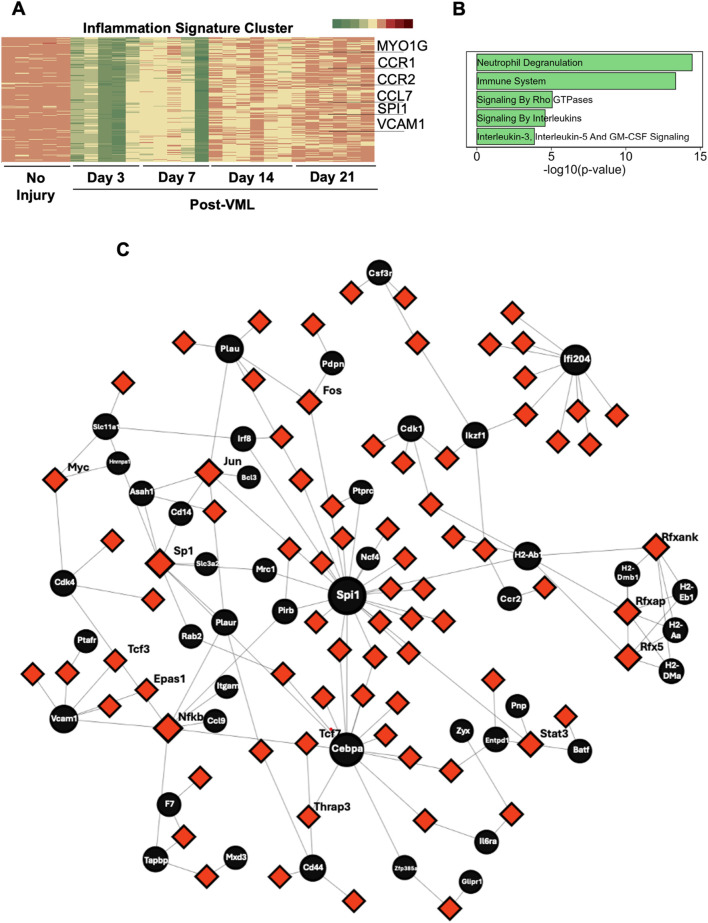
Inflammatory signaling cluster reveals gene upregulation and *Spi1* transcription factor gene networks. **(A)** Heatmap of all genes belonging to the inflammation cluster. **(B)** Gene enrichment bar plot of genes in inflammation cluster. **(C)** Gene-transcription factor networks of genes (black circles) in the inflammation cluster and their connection with any known transcription factors.

The inflammation-related genes cluster was another prominent cluster showing sustained upregulation over 21 days (Figure 1A). The temporal pattern of this gene cluster exhibited a sharp increase in gene expression after VML, followed by a stepwise reduction over time. Some of the key genes in this temporal cluster that exhibited >3-fold change increase on day 3, relative to day 0, were *CCR1*, *CCR2*, *SPI1,* and *VCAM1* (Figure 3A)*,* which are involved in cell migration and recruitment of immune cells to the injury site in the skeletal muscle (Yahiaoui et al., 2008; Choo et al., 2017; Wang et al., 2022). On day 21, genes like *IL3RA*, *CSF2RA,* and *SGPL1* remained elevated with an average 3-fold increase compared to non-injury control. Although inflammation pathways are necessary to initiate the regeneration response, chronically elevated gene expression has been shown to have adverse effects that lead to cell death or fibrosis (Larouche et al., 2018). Gene enrichment analysis using the Reactome 2022 database revealed significant enrichment in the categories of Neutrophil Degradation (37 genes in total; P < 3.5 × 10^−15^) including *SLC11a1* and *NFAM1;* and Innate Immune System (74 genes in total; P < 4.5 × 10^−14^) including *CSF3R, CCR2* (Figure 3B). Intriguingly, multiple gene sets related to Rho GTPases were also significantly enriched in this cluster of 28 genes on day 21 (P < 8.7 × 10^−07^), including *ARPC1B, DBN1,* and *SPC25*). Rho GTPases are essential players by activating satellite cells (Rodriguez-Fdez and Bustelo, 2021), so their sustained upregulation suggests compensatory activation of regeneration-related pathways to counteract inflammatory processes (Figure 3B).

In order to identify central regulators of this sustained inflammation, we focused on early time point targets using the transcription factor gene network, which revealed *SPI1* (>48-fold change on day 3) to be one of the most connected nodes for the gene regulatory network (Figure 3C). *SPI1* is a transcription factor that controls the development of myeloid and B-lymphoid cells, which are shown to be involved in fibrosis and muscle atrophy in skeletal muscle (Wang et al., 2022). Furthermore, within the transcription factor-gene network, *SPI1* is closely related to *NCF4* and *CEBPA*, both of which have a role in macrophage activation and infection response (Lee et al., 2014). Interestingly, the transcription factor (red nodes) that regulated most of the genes in this inflammation cluster (black nodes) was *SP1* (>2.2 fold change on day 3, compared to day 0), which is consistent with what was observed with ECM-related clusters. Consequently, *SP1* could be a novel target for regulating inflammation after VML.

### Mitochondria function and oxidative metabolism show sustained downregulation after VML injury, with enrichment in oxidative phosphorylation and adipogenesis

**FIGURE 4:**
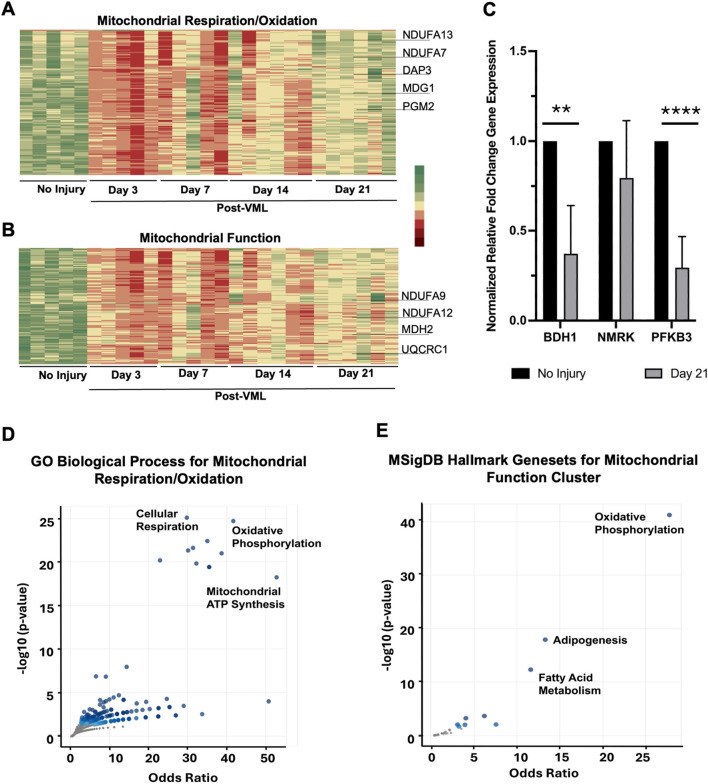
Mitochondria function and oxidative metabolism clusters showed downregulation after VML injury. **(A,B)** Heatmap of all genes belonging to the mitochondrial respiration/oxidation cluster **(A)** and mitochondrial function cluster **(B)**, respectively. **(C)** qPCR gene expression validation of differentially expressed genes showing significant downregulation after 21 days of VML, compared to no injury control group (*n* = 4–5, **P < 0.01, ****P < 0.0001). **(D,E)** Gene enrichment volcano plots of genes in the mitochondrial respiration/oxidation cluster **(D)** and mitochondrial function cluster **(E)**, respectively.

In contrast to the gene clusters that showed upregulation patterns, two gene clusters with mitochondria function and oxidative metabolism identities were characterized by significant downregulation immediately after injury, followed by progressive increases in gene expression over time (Figures 4A,B). Notably, these two clusters consisted of approximately 400 genes with incomplete recovery, with >2.2-fold change downregulation on day 21, compared to pre-injury levels. These genes included 27 out of 40 subunits of *NDUF* proteins that are a part of NADH, a ubiquinone oxidoreductase core in the inner membrane of mitochondria (Figures 4A,B). The DEGs associated with specific metabolic markers were further validated by qPCR, namely, *BDH1* (ketone body metabolism, −2.8 log Fold Change at Day 21 post-VML), *NMRK* (energy metabolism, −2.4 logFC at Day 21 post-VML) and *PFKFB3* (regulating glycolysis, −2.7 logFC at Day 21 post-VML). Similar downregulation in mitochondrial or metabolism-related genes was observed using qPCR for *BDH1 and PFKFB3* (Figure 4C, **P < 0.01 and ****P < 0.0001).

Gene set enrichment analysis of these two clusters revealed numerous mitochondrial function-related or cellular metabolism-related gene sets. In particular, oxidative phosphorylation (P = 1.88 × 10^−25^) and cellular respiration (P = 7.2 × 10^−23^) were the most enriched gene sets for the mitochondrial respiration/oxidation cluster (Figure 4D). Among the DEGs, These enriched gene sets included multiple *NDUF* and *COX* genes. *COX* encodes enzymes that convert arachidonic acid to prostaglandins, which has been shown to upregulate muscle regeneration (Bondesen et al., 2004). The sustained downregulation of the *COX* genes in the context of mitochondrial respiration suggests a role for cellular respiration in muscle regeneration.

In the mitochondrial function gene cluster, oxidative phosphorylation was also found to be enriched (P = 2.9 × 10^−41^), including several versions of malate dehydrogenase (*MDH*) enzymes, along with adipogenesis genes (P = 3 × 10^−18^) such as *VEGFb* and *ALDOA*) (Figure 4E). Although not reported in the context of VML, a knockdown of *VEGFb* has been linked to adipose browning and a decrease in muscle growth via energy-dependent pathways in mice, signifying the importance of adipogenesis in muscle regeneration (Ling et al., 2022). In addition, fatty acid metabolism (P = 9.0 × 10^−13^) as characterized by *MLYCD,* along with glycolysis-related genes (P = 0.02), were enriched in the mitochondrial function temporal cluster, signifying an overall reduction of cellular metabolism in the muscle after VML injury (Li et al., 2021). In summary, a sustained decrease in cellular respiration, metabolism, and adipogenesis was regulated by mitochondrial machinery after VML induction.

In addition, we observed another temporal cluster with a notable downregulation pattern at earlier time points corresponding to muscle development (Supplementary Figures S1A–C). This cluster was characterized by immediate downregulation, with a gradual increase in gene expression after 7 days (Supplementary Figure S1B). Some of the genes in this cluster consisted of cytoskeletal markers such as *MYL1* and *TTN* that are critical for proper muscle structure (Supplementary Figure S1A). This suggests that even though genes corresponding to structural components of the muscle do return to non-injury levels, the functionality of the regenerated muscle remains dysregulated as evident in the significant reduction in mitochondrial function and energy metabolism. This finding is consistent with VML inducing irreversible impairment in muscle function in preclinical VML models (Shayan and Huang, 2020; Clark et al., 2022; Hu et al., 2025).

### RAS pathway, mTOR pathway and neuromuscular genes

**FIGURE 5:**
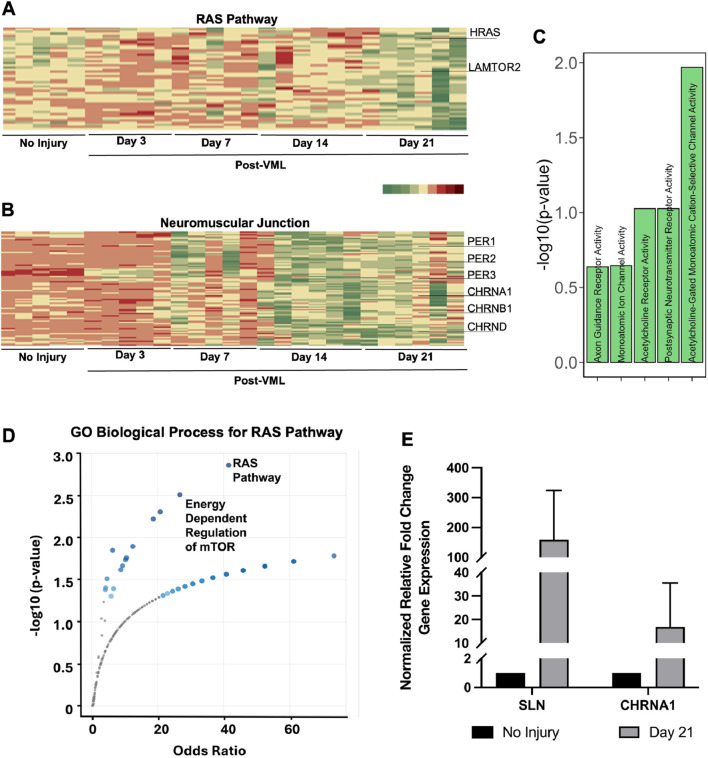
RAS pathway, mTOR pathway and neuromuscular genes show non-uniform gene expression temporal profiles. **(A,B)** Heatmap of all genes belonging to the RAS pathway cluster **(A)** and neuromuscular junction cluster **(B)**, respectively. **(C)** Gene enrichment bar plot of genes in neuromuscular junction cluster. **(D)** Gene enrichment volcano plots of genes in RAS Pathway cluster. **(E)** qPCR gene expression validation of differentially expressed genes showing trends of upregulation after 21 days of VML, compared to no injury control group (*n* = 4–5).

Besides the first two patterns of genes showing upregulation or downregulation patterns, we also observed clusters that demonstrated irregular gene expression patterns. In particular, the RAS/mTOR pathway and Neuromuscular junctions cluster were downregulated initially after injury but upregulated beyond pre-injury levels by 21 days after VML (Figures 5A–D). In the RAS/mTOR pathway cluster, a marked increase in *HRAS* and *LAMTOR2* (both activators of MAPK and MTOR signaling) was observed, particularly on day 21 (Figure 5A). Since this pathway modulates metabolism in muscle, this upregulation pattern could be in response to defective mitochondrial function (Yoon, 2017). Gene enrichment analysis also revealed significant enrichment of the RAS/mTOR pathway cluster to RAS pathway genes (P = 0.001) and Energy Dependent Regulation of mTOR (P = 0.003) in the Reactome database (Figure 5D). In addition, the cluster associated with neuromuscular junction genes showed marked upregulation only after 14 days (Figure 5B). In particular, this cluster included multiple subunits of cholinergic receptors, such as *CHRNA1*, *CHRNB1*, and *CHRND*. This gene cluster also had significant enrichment in gene sets such as Acetylcholine-Gated Channel Activity (P = 7.0 × 10^−05^) and Axon Guidance Receptor Activity (Figure 5C, P = 0.02). These findings were additionally verified by quantification of mRNA for *CHRNA1* and *SLN* (sarcolipin, calcium ion channel in muscles). Both *CHRNA1* and *SLN* were upregulated at 21 days after VML by 16 and 185-fold, respectively, compared to no VML control (Figure 5D). This finding suggests an association of defective muscle regeneration with impaired neuromuscular junction interactions.

### Cytokine proteomics show sustained elevation of pro-inflammatory factors

**FIGURE 6:**
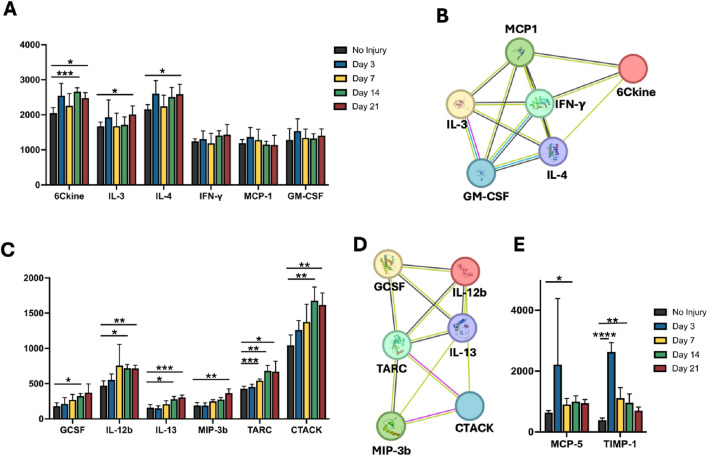
Validation of inflammatory transcriptomic signature using cytokine proteomics. Proteomic analysis of inflammation-related cytokines on muscle lysates at each time point. **(A)** The first cluster was categorized as cytokines that demonstrated a significant increase in level over time. These cytokines consisted of those that regulate the inflammatory and repair phases of muscle regeneration. **(B)**. Interactions among the proteins within cluster **(A)** that guide muscle healing processes. **(C)** Additional cytokines with persistent protein expression increases are associated with the management of inflammation, immune cell migration, and tissue repair. **(D)** The interactions detected in this protein cluster relate to immune cell recruitment, signaling, and macrophage activation. **(E)** Cytokines were identified to have a significant decrease after induction of VML. *P < 0.05, **P < 0.01, ***P < 0.001, ****P < 0.0001.

To validate the findings of a sustained inflammatory transcriptomic signature, we performed a temporal proteomic analysis of 32 inflammation-related cytokines in muscle lysates at each time point. Among them, 24 cytokines demonstrated significant differences in protein levels and were categorized into clusters as well (P < 0.05). The first cluster was categorized as cytokines, with an increase in level by day 21. These cytokines consisted of those that regulate the inflammatory and repair phases of muscle regeneration (Figure 6A), including 6Ckine that recruits necessary immune cells (day 0 vs. day 14, ***P = 0.0008; day 0 vs. day 21, *P = 0.015); IL-3 that supports the proliferation of hematopoietic cells and influences immune cell function (day 0 vs. day 21, *P = 0.049), and IL-4 that promotes a tissue-repairing environment (day 0 vs. day 21, *P = 0.02) (Fujita et al., 2022; Podolska et al., 2024). These cytokines did not revert to pre-injury expression levels by day 21, suggesting the persistence of immunomodulatory signals to support tissue remodeling to the affected muscle tissue. The sustained elevated levels of these inflammatory cytokines suggest dysregulation in the tissue recovery process. We further analyzed the interactions among the proteins within this cluster (Figure 6B) that guide muscle healing processes. The key player identified in this protein cluster was interferon-γ (IFN-γ), whose upregulation stimulates a response from all other cluster cytokines and is essential for early pro-inflammatory response (Gordon and Taylor, 2005). 6Ckine levels are directly regulated by IFN- γ and IL-4, while being indirectly regulated by IL-3 through its enhancement of the hematopoietic response (de Bruin et al., 2014; Naradikian et al., 2016). Furthermore, IL-3 enhances the roles of MCP-1 and GM-CSF due to the immune cell recruitment inherent in its increased expression (Barnes, 2008; Zarei et al., 2009). Therefore, it is possible that the dysregulated cytokine expression levels prohibit effective participation in the inflammatory response to VML.

Additional cytokines with statistically significant high protein expression over time were associated with the management of inflammation, immune cell migration, and tissue repair (Figure 6C, P < 0.0.05). In normal healing processes, the early inflammatory phase is driven by GCSF and IL-12B, while IL-13, MIP-3β, CTACK, and TARC help transition the response to a repair phase and ensure proper resolution of inflammation (Koh and DiPietro, 2011; Oishi and Manabe, 2018). The interactions detected in this protein cluster (Figure 6D) relate to immune cell recruitment, signaling, and macrophage activation. During muscle repair and remodeling processes, MIP-3β, TARK, and CTACK are modulated by IL-13 for Th2 and T cell recruitment to the injury site (Wu et al., 2022). IL-12B and IL-13 levels balance each other by promoting Th1 and Th2 responses, respectively. GCSF and IL-13 facilitate macrophage polarization (Wang et al., 2014; Atri et al., 2018). The protein interaction network suggests that the balance and timing of these interactions prohibit effective muscle regeneration in the setting of VML. Although less prevalent, two cytokines were identified to have a significant decrease in cytokine levels, including MCP-5, which regulates inflammatory responses through the attraction of immune cells, and TIMP-1, which interacts with T cells and facilitates Th2 phase for tissue repair (Knight et al., 2019; Kokubo et al., 2022; Lee and Kim, 2022) (Figure 6E). Together, this cytokine analysis demonstrates time-dependent changes in protein levels that may point to dysregulation of inflammatory processes that impede effective recovery after VML.

### VML transcriptional signature

**FIGURE 7:**
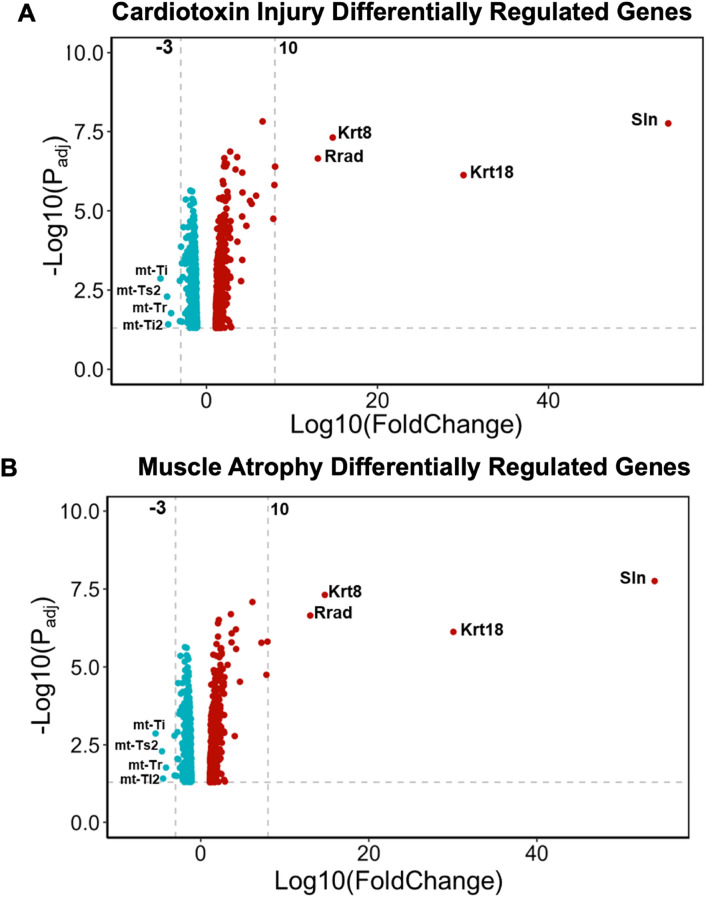
Characterization of a unique VML gene signature. **(A)** Volcano plot of uniquely differentially expressed genes in our VML transcriptomics dataset, compared to mouse cardiotoxin-induced muscle injury dataset. **(B)** Volcano plot of uniquely differentially expressed genes in our VML transcriptomics dataset compared to human muscle atrophy dataset.

Based on these findings, we further sought to identify a signature that characterizes the transcriptional signature of a non-healing muscle injury model like VML to that of a healing model of cardiotoxin-induced muscle injury. In particular, we compared the DEGs on day 21 between VML injury with that of cardiotoxin-induced muscle injury (Oprescu et al., 2020). Intriguingly, we identified a unique signature of VML-specific genes characterized by the greatest fold change in the unique DEGs (Figure 7). The unique VML signature was associated with the upregulation of *SLN* (Sarcolipin; thermogenic and metabolism regulator), *KRT8* (Keratin 8, cytoskeletal fibrotic marker), *KRT18* (Keratin 18; cytoskeletal fibrotic marker) and *RRAD* (Ras-related GTPase). Concomitantly, the VML signature was also characterized by the downregulation of mitochondrial genes, namely, *MT-TI*, *MT-TI2*, *MT-TS2*, and *MT-TR*. These data suggest a unique signature associated with VML that is not associated with fully healing toxin injury models. To further explore this signature, we also compared our findings with a clinical dataset from hospitalized patients having severe skeletal muscle wasting patients (Llano-Diez et al., 2019). Similar findings were found where the VML signature was marked by upregulation of *SLN*, *KRT8*, *KRT18* and *RRAD*, concomitant with a significant downregulation in specific mitochondrial genes. Together, this analysis reveals unique gene expression signatures associated with VML, in contrast to healing muscle injuries or atrophic muscle.

## Discussion

This work elucidated the temporal transcriptional landscape of tissue remodeling after VML in a mouse model. Bulk RNAseq and time-series analysis were performed to identify temporal gene clusters based on temporal expression patterns reflecting sustained upregulation, sustained downregulation, or those with irregular expression patterns. The salient findings are that: (1) gene clusters with sustained upregulation were generally associated with ECM remodeling and inflammation, with strong cell cycle and *SP1* transcription factor gene network interactions; (2) gene clusters with sustained downregulation were associated with mitochondrial function and metabolism with enrichment in oxidative phosphorylation and adipogenesis; (3) by examining gene clusters with mixed regulation patterns, we identified a putative role of RAS/mTOR pathway in later stages of recovery, possibly as a compensatory mechanism due to the loss of mitochondrial function.

Complementary to transcriptomic profiling, cytokine profiling also revealed the dysregulation of inflammatory factors. The balance and timing of cytokine expression levels are essential for effective muscle regeneration. Since 6Ckine and IFN-γ are heavily involved in the initial inflammatory phase, these factors might act by recruiting M1 macrophages and T cells to clear tissue damage initially. However, the observed sustained upregulation of IFN-γ indicates a chronic inflammatory stage that contributes to fibrosis and eventual impairment of the regenerative process. These pro-inflammatory factors are counteracted by IL-13, TARC, and MIP-3β which potentially act to shift the environment to one that is repair-driven and anti-inflammatory.

Our findings concur with other reports of transcriptomic analysis after VML injury. In a VML canine model, the authors reported similar roles for collagen 1 and fibronectin in sustained fibrosis and a significant role in macrophage activation. Additionally, spatial transcriptomics of a VML injury model reported a fibrotic zone with similar ECM composition (*FN1, COL1A1, COL6A1,* AND *COL4A2, LAMA2*) and inflammatory markers (*CCR1, NCAM1*) (Larouche et al., 2023), as did our findings show. Other reports of VML injury transcriptomics up to 14 days after injury report similar findings regarding sustained fibrosis and inflammation despite an increase in myogenesis (Nuutila et al., 2017; Aguilar et al., 2018).

Despite some similarities in findings with prior literature, this study extends the fundamental understanding of VML pathological remodeling, using distinct temporal patterns to identify pathways that deviate from normal pre-injury levels. In particular, we showed mitochondrial dysregulation and decreased cellular respiration in the injured muscle, even after 21 days of injury. In addition, we also demonstrated a novel putative role of insufficient adipogenesis via *VEGFB* pathway, suggesting insufficient numbers of FAP (Fibro-adipo progenitor) cell activation that accompanies muscle regeneration (De Micheli et al., 2020). Owing to our non-biased approach in delineating these temporal patterns, we also sought to find key regulators of these temporal clusters using transcription factor-gene networks. Specifically, we found key regulators for sustained fibrosis and inflammation. One of the potential key targets identified from our analysis is the *SP1* transcription factor. *SP1* is linked to pro-fibrosis, an inflammation in many diseases such as muscular dystrophy and atherosclerosis (Vinals et al., 1997). In skeletal muscle, *MYOD1* (a muscle regeneration transcription factor) has been shown to downregulate *SP1*, which aligns with the hypothesis of targeting *SP1* for reducing fibrosis and inflammation, leading to better repair (Vinals et al., 1997). Another therapeutic target derived from our work is the mitochondria. The significant role of mitochondrial function and oxidative phosphorylation is impactful and paradigm-shifting for VML injury therapeutics. Drugs targeting the mitochondria can potentially treat muscular conditions of VML. Future work to delineate the role of *Sp1* or mitochondrial targets is warranted in the setting of VML.

A limitation of the current work is that it does not address concerns of potential compensatory hypertrophy in the context of VML. For example, one group reported that the extensor digitorum longus muscles of the surgically removed TA muscle was higher in muscle mass and cross-sectional, compared to the ipsilateral uninjured leg (Freeman et al., 1982). However, other studies were inconclusive in showing compensatory hypertrophy of other muscle groups (Wu et al., 2012). Therefore, studies to further understand the extent of compensatory hypertrophy as a consequence of VML requires further attention in the future.

In summary, the temporal kinetics of transcriptional and proteomic signatures were evaluated using time-series analysis in a mouse VML model. Our findings demonstrated dysregulated muscle transcriptional signatures with patterns of sustained upregulation, downregulation, or those with irregular patterns. Geneset enrichment analysis revealed dysregulated pathways associated with ECM remodeling, inflammation, mitochondrial function, and metabolism while identifying a potential role of *Sp1* transcription factor gene network interactions in mediating the process. These findings reveal the temporal kinetics of pathological tissue remodeling and can be applied toward the identification of novel gene targets that may augment muscle regeneration.

## Data Availability

The datasets presented in this study can be found in online repositories. The names of the repository/repositories and accession number(s) can be found in the article/Supplementary Material.

## Supplementary material

SUPPLEMENTARY FIGURE S1
Muscle development cluster shows genes associated with muscle development after induction. **(A)** Heatmap of genes associated with muscle development after induction of VML for up to 21 days. **(B)** Line graph of genes in the muscle development cluster over time after injury. The black lines within each histogram denote genes that were significantly differentially expressed at one or more time points, whereas the red lines indicate genes that were significantly differentially expressed on day 21, compared to the control (non-injured) samples (P < 0.05). **(C)** Gene enrichment bar plots of genes in the muscle development cluster.
