# Supplementary figures and images for: Temporal dynamics of gene and protein signatures following volumetric muscle loss
Source: Front Cell Dev Biol. 2025 Jul 3;13:1606609. doi: 10.3389/fcell.2025.1606609 (PMC12268354; doi:10.3389/fcell.2025.1606609)

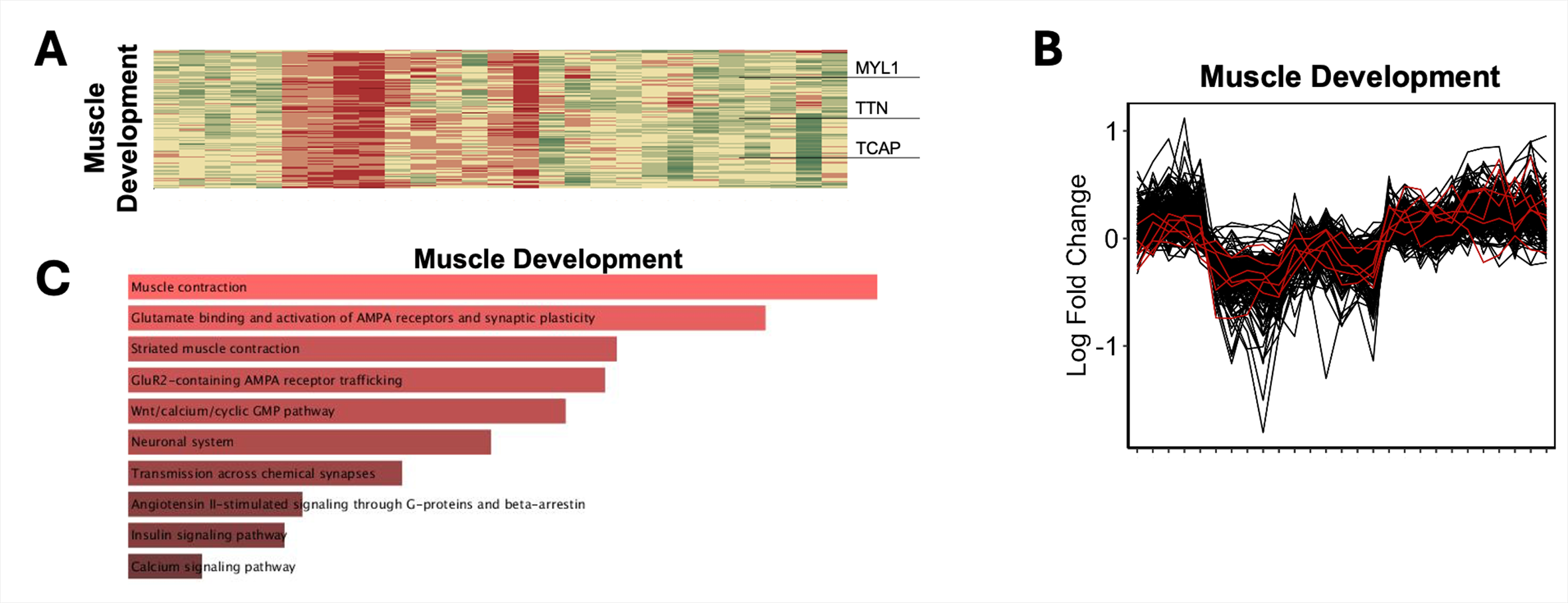

Supplement: Supplementary file 1 [file Image1.tif]
